# *Solanum macrocarpon* L. Ethanolic Leaf Extract Exhibits Neuroprotective and Anxiolytic Effects in Scopolamine-Induced Amnesic Zebrafish Model

**DOI:** 10.3390/ph18050706

**Published:** 2025-05-09

**Authors:** Ion Brinza, Corina Guliev, Ibukun Oluwabukola Oresanya, Hasya Nazli Gok, Ilkay Erdogan Orhan, Lucian Hritcu

**Affiliations:** 1 Faculty of Sciences, Lucian Blaga University of Sibiu, 550024 Sibiu, Romania; ion.brinza@ulbsibiu.ro (I.B.); corinaioana.guliev@ulbsibiu.ro (C.G.); 2 Department of Pharmacognosy, Faculty of Pharmacy, Gazi University, 06330 Ankara, Türkiye; ayoola.ibukun@gmail.com (I.O.O.); hasyaekin@gazi.edu.tr (H.N.G.); 3 Department of Pharmacognosy, Faculty of Pharmacy, Lokman Hekim University, 06510 Ankara, Türkiye; ilkay.erdoganorhan@lokmanhekim.edu.tr; 4 Department of Biology, Faculty of Biology, Alexandru Ioan Cuza University of Iasi, 700506 Iasi, Romania

**Keywords:** memory, acetylcholinesterase, oxidative stress, ADMET prediction, PASS analysis, phytochemicals

## Abstract

**Background/Objectives**: *Solanum macrocarpon* L. has been studied for its neuroprotective potential and memory-enhancing properties. Research suggests that bioactive compounds, including flavonoids, alkaloids, and phenolics, contribute to its cognitive benefits. These compounds may help protect against oxidative stress, neuroinflammation, and cholinergic dysfunction factors in memory impairment. This study was undertaken to investigate the effects of *S. macrocarpon* ethanolic leaf extract (SMEE) on the memory, anxiety-like behavior, and brain antioxidant status of scopolamine (SCOP, 100 μM)-induced amnesic zebrafish (*Danio rerio*) and thus to understand its possible mechanism of action. **Methods**: Adult zebrafish (*n* = 100) were divided into two cohorts (±SCOP) of five experimental groups: (I) control; (II) galantamine (GAL, 1 mg/L), serving as a positive control for both behavioral and biochemical assessments; (III–V) three groups treated with SMEE (1, 3, and 6 mg/L); (VI) scopolamine (SCOP, 100 μM); (VII) SCOP (100 μM) combined with GAL (1 mg/L); and (VIII–X) three groups treated with SCOP (100 μM) plus SMEE (1, 3, and 6 mg/L). The treatment lasted 23 days and amnesia was induced by a single dose of SCOP (100 μM) before testing. **Results**: The phenolic characterization from the samples was performed by using HPLC-PDA chromatography. Following HPLC analysis, an in silico pharmacokinetic evaluation was conducted using the ADMET model to investigate the pharmacological and toxicological profiles of the identified compounds. Spatial memory was evaluated through the Y-maze and novel object recognition (NOR) tests, while anxiety-like behavior was assessed using the novel tank diving test (NTT), novel approach test (NAT), and light–dark test (LDT). The zebrafish were euthanized, and homogenates of isolated brain samples were assayed for acetylcholinesterase (AChE) activity and brain antioxidant markers. The HPLC analysis revealed that the main major compounds in the extract were chlorogenic acid and rutin, both recognized for their significant antioxidant properties. **Conclusions**: SMEE enhanced memory by inhibiting AChE, alleviated SCOP-induced anxiety-like behavior, and significantly decreased oxidative stress markers. These findings support the potential role of SMEE in counteracting SCOP-induced cognitive and behavioral dysfunctions, related to dementia conditions.

## 1. Introduction

Alzheimer’s disease (AD) is an irreversible neurodegenerative disorder characterized by progressive deterioration of cognitive and functional abilities, manifested by episodic memory loss, speech difficulties, neuropsychiatric symptoms, and, in advanced stages, premature death [1]. As the most common form of dementia, AD accounts for 60 to 80% of all cases worldwide, currently affecting approximately 25 million people. It is estimated that, with increasing life expectancy, the number of diagnosed patients will exceed 115 million by 2050 [2].

Pathologically, AD is characterized by the extracellular deposition of β-amyloid peptide (Aβ) plaques and the intracellular accumulation of neurofibrillary tangles formed by hyperphosphorylated tau protein. These processes contribute to neuronal degeneration and progressive deterioration of brain functions [3]. Current evidence also indicates that oxidative stress and mitochondrial dysfunction play a key role in the etiopathogenesis of AD and other neurodegenerative diseases, such as Parkinson’s disease (PD), amyotrophic lateral sclerosis (ALS), and Huntington’s disease (HD). Evidence from research demonstrates that AD is linked to a decrease in complex IV activity and an elevation in oxidative stress levels. In contrast, PD is associated with reduced complex I activity, and HD with impaired complex II function and increased oxidative stress in the cortex. In the case of ALS, familial forms are attributed to mutations in the enzyme Cu/Zn superoxide dismutase (SOD1), while the sporadic variant of the disease is associated with increased oxidative stress [4]. Another pathophysiological mechanism involved in AD is the damage to the cholinergic system, especially of neurons in the basal ganglia, a phenomenon that led to the formulation of the cholinergic hypothesis regarding the cognitive deficits characteristic of the disease. However, recent studies support the idea of an interconnection between the different proposed pathogenic mechanisms. For example, exposure of cholinergic neurons to Aβ peptides has been associated with cytotoxic effects, and their activation can influence the metabolism of amyloid protein and the phosphorylation of tau protein. Furthermore, it has been found that acetylcholinesterase (AChE) interacts with presenilin 1, the catalytic subunit of γ-secretase, mutually regulating their expression and activity [5].

Despite advances in understanding the mechanisms involved in the development of AD, available treatments are limited in efficacy and may have significant adverse effects. Therefore, current research is exploring therapeutic alternatives, including the use of bioactive compounds, as possible strategies to slow disease progression. Although the relationship between nutrition and AD is still insufficiently explored, preclinical studies suggest that certain natural approaches, based on the principles of neurohormesis (phenomenon in which moderate exposure to stressors activates neuronal protection mechanisms, stimulating synaptic plasticity and reducing the risk of neurodegenerative diseases), could modulate pathological mechanisms and influence the course of the disease [2,6].

Zebrafish (*Danio rerio*) is a valuable vertebrate model in neurobiological research, used to investigate the mechanisms involved in brain development and various neurological disorders. Due to genetic conservation with mammals, this experimental model allows for testing the effects of compounds on cognitive function and behavior [7]. It retains homologous vertebrate brain subdivisions, such as the thalamus, optic tectum, and cerebellum, along with conserved neurotransmitter systems, including gamma-aminobutyric acid (GABA), glutamate, histamine, dopamine, acetylcholine (ACh), and serotonin [8]. Pharmacological similarity is also evidenced by the comparable effects of psychoactive compounds on sleep regulation in zebrafish and mammals. Approximately 80% of human risk genes associated with neurodevelopmental disorders have orthologs in this model, indicating substantial genetic conservation. In addition, fundamental neural circuits involved in essential behaviors are maintained, which strengthens the applicability of zebrafish for investigating the neurobiological mechanisms of neurodevelopmental disorders [9]. Supplementary to that, zebrafish are used to test the effects of natural bio-compounds on neurocognitive signaling pathways. Exposure to certain natural extracts, polyphenols, and flavonoids influences critical molecular factors, such as CREB, BDNF, NGF, and CaMKII, with potential for neuroprotection and stimulation of synaptic plasticity [10].

*Solanum macrocarpon* is a tropical perennial plant belonging to the Solanaceae family, used both in food and in traditional medicine, especially in regions of West and Central Africa. Its leaves and fruits are consumed in the form of soups and sauces, with a characteristic bitter taste, and the plant is also used as animal feed [11]. In addition to its nutritional value, *S. macrocarpon* is of scientific interest due to its bioactive compounds, such as flavonoids, alkaloids, saponins, and tannins, which confer various pharmacological properties [12]. Among the reported therapeutic effects are antioxidant activity, attributed to the high content of phenolic compounds, which suggests a potential role in improving fertility, positively influencing parameters such as sperm mobility and morphology [11]. It also has lipid-lowering and hepatoprotective effects, which indicate the possibility of its use in the prevention of metabolic disorders [13]. Moreover, its laxative, anthelmintic, and hypotensive properties mean it has traditionally been used in the treatment of gastrointestinal and cardiovascular conditions [14]. Despite its long-standing use in traditional medicine, the specific effects of *S. macrocarpon* on human health, as well as its toxicological profile, are not fully elucidated.

This study aims to analyze the chemical composition of the ethanolic extract of *S. macrocarpon* leaves (SMEE) and to investigate its pharmacological effects in an experimental model of scopolamine (SCOP)-induced cognitive deficit in zebrafish. SCOP, a muscarinic acetylcholine receptor antagonist, is widely used to induce amnesia in zebrafish (*Danio rerio*), serving as a model for studying cognitive impairments and neurodegenerative disorders such as AD. By blocking cholinergic signaling, SCOP disrupts learning and memory processes, leading to deficits in spatial and associative tasks. Zebrafish exposed to SCOP show impaired performance in cognitive assays like the Y-maze and novel object recognition test, reflecting deficits in spatial and non-spatial memory [15]. Additionally, SCOP increases anxiety-like behavior and oxidative stress markers, mimicking pathological conditions observed in neurodegeneration [16]. Although *S. macrocarpon* has been traditionally used for its antioxidant, hepatoprotective, and anti-inflammatory properties [17,18], there is a lack of studies evaluating its effects on cognitive function and behavior in validated animal models of neurodegeneration. To the best of our knowledge, no previous research has examined the neuroprotective or anxiolytic potential of SMEE in a SCOP-induced zebrafish model of cognitive impairment. Given the translational value of the zebrafish model for studying cholinergic dysfunction and oxidative stress in AD-like conditions, this study aimed to bridge that gap. Therefore, we investigated whether SMEE could ameliorate memory deficits, anxiety-like behavior, and biochemical markers of neurodegeneration, offering new insights into its therapeutic potential against dementia-related pathologies.

## 2. Results

### 2.1. Chemical Composition of SMEE

**Figure 1:**
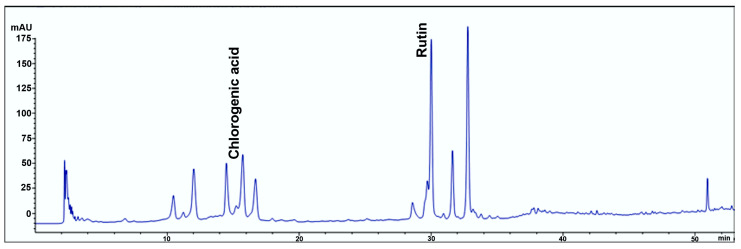
HPLC chromatogram of SMEE at 320 nm.

The separation of phenolic compounds in the SMEE extracts is shown in Figure 1, and their quantified contents are provided in Table 1. HPLC analysis confirmed the presence of chlorogenic acid and rutin among the tested phenolic compounds.

In the SMEE extract, chlorogenic acid was identified as the most abundant phenolic compound (25.46 ± 0.10 mg/g), followed by rutin (7.55 ± 0.10 mg/g).

### 2.2. Evaluation of the Pharmacological Properties

The pharmacokinetic and toxicological properties of the analyzed compounds (SCOP, GAL, chlorogenic acid, and rutin) regarding their absorption, distribution, metabolism, excretion, and toxicity (ADMET) are presented in Table 2.

The ADMET pharmacokinetic predictions indicate that SCOP and GAL exhibit high intestinal absorption (72.626% and 94.994%, respectively), whereas chlorogenic acid (36.377%) and rutin (23.446%) have lower absorption rates, suggesting limited oral bioavailability for the latter. Skin permeability is low for all analyzed compounds (log Kp < −2.5), indicating reduced transdermal penetration. Regarding the interaction with P-glycoprotein, both chlorogenic acid and rutin are substrates of this efflux protein, which may influence their cellular efflux and bioavailability, whereas SCOP and GAL do not inhibit this efflux protein.

VDss is relatively high for GAL (0.89 log L/kg) and rutin (1.633 log L/kg), suggesting an extensive distribution within the body, while SCOP and chlorogenic acid show moderate values. The blood–brain barrier (BBB) permeability analysis indicates that neither chlorogenic acid nor rutin can efficiently cross the BBB, unlike SCOP and GAL, which showed limited permeability. Additionally, the ability of these compounds to penetrate the CNS is very low for chlorogenic acid and rutin (log PS < 3), suggesting a predominantly peripheral action.

SCOP and GAL are substrates of the CYP3A4 enzyme, indicating potentially extensive hepatic metabolism, whereas chlorogenic acid and rutin are not metabolized via this enzyme. None of the analyzed compounds inhibit CYP1A2, reducing the risk of drug interactions associated with this enzyme.

GAL exhibits a higher total clearance rate (0.991 log mL/min/kg) compared to SCOP (1.096 log mL/min/kg), while chlorogenic acid and rutin have significantly lower clearance rates, suggesting slower elimination. Among the analyzed compounds, only GAL is a substrate for the renal transporter OCT2, which may influence its renal excretion.

The toxicological profile shows significant variations among the compounds. GAL presents the highest risk of cardiotoxicity (hERG blockers 0.458), while chlorogenic acid and rutin have significantly lower risks. GAL and SCOP show the highest scores for hepatotoxicity, whereas rutin has an increased risk of drug-induced liver toxicity (0.937) and genotoxicity (0.868). Additionally, GAL and SCOP exhibit moderate neurotoxicity, in contrast to chlorogenic acid and rutin, which have very low scores in this regard.

### 2.3. In Silico Prediction of Biological Activity of SMEE Compounds

**Figure 2:**
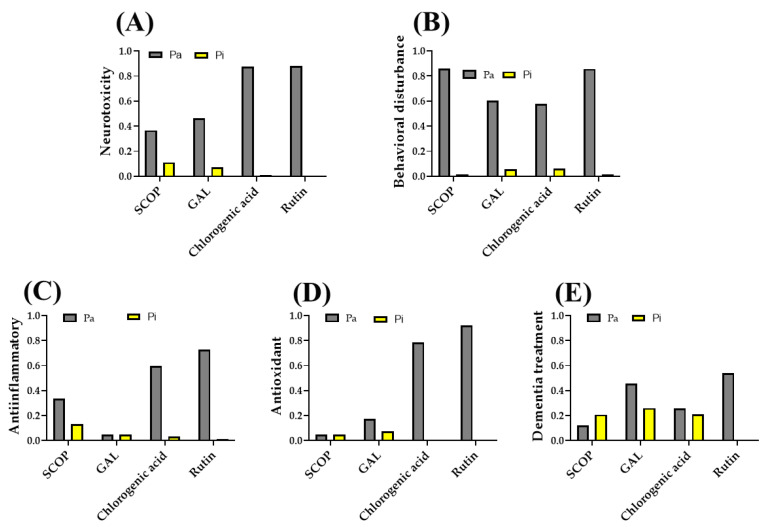
PASS prediction outcomes for SCOP, GAL, chlorogenic acid, and rutin, assessing their potential for (**A**) neurotoxicity, (**B**) behavioral disturbance, (**C**) anti-inflammatory activity, (**D**) antioxidant activity, and (**E**) pharmacological relevance in dementia treatment. Pa—probability of activity; Pi—probability of inactivity.

The interpretation of the data regarding the biological activities of the analyzed compounds (SCOP, GAL, chlorogenic acid, and rutin) provides insight into their pharmacological potential, based on the probability of activity (Pa) and the probability of inactivity (Pi). The data suggest that rutin and chlorogenic acid have the highest potential risk of adverse effects, particularly concerning inflammation and neurotoxicity. Chlorogenic acid (Pa = 0.874) and rutin (Pa = 0.882) exhibit the highest neurotoxicity risk scores, while GAL (Pa = 0.463) and SCOP (Pa = 0.367) have lower values (Figure 2A).

Regarding the induction or triggering of behavioral disorders, SCOP (Pa = 0.861) and rutin (Pa = 0.857) show the highest risk, followed by GAL and chlorogenic acid (Figure 2B). However, rutin (Pa = 0.728) and chlorogenic acid (Pa = 0.598) demonstrate a higher anti-inflammatory potential compared to SCOP (Pa = 0.338) and GAL (Pa = 0.05) (Figure 2C). In terms of antioxidant activity, rutin (Pa = 0.923) and chlorogenic acid (Pa = 0.785) are the most promising, whereas SCOP and GAL show significantly lower values (Figure 2D). These results denoted a high potential of rutin and chlorogenic acid in reducing oxidative stress and inflammation. Additionally, rutin exhibited the highest probability of activity (Pa = 0.541) for dementia treatment, followed by GAL (Pa = 0.458) and chlorogenic acid (Pa = 0.258). SCOP has the lowest probability of efficacy (Pa = 0.121), suggesting a limited impact in this field (Figure 2E).

### 2.4. Safety Assessment of Chronic Exposure to SMEE

Chronic administration of SMEE (1, 3, and 6 mg/L) did not result in any mortality or observable signs of toxicity in the zebrafish. Fish were monitored daily for visible morphological abnormalities (e.g., discoloration, fin damage, body curvature), behavioral changes (e.g., hypo/hyperactivity, erratic movement, loss of balance), and physiological stress responses (e.g., surface gasping, feeding inhibition). No adverse effects were observed across any group, indicating that the extract was well-tolerated under chronic exposure conditions.

### 2.5. Effects on Anxiety-like Behavior in NTT, NAT, and LDT

**Figure 3:**
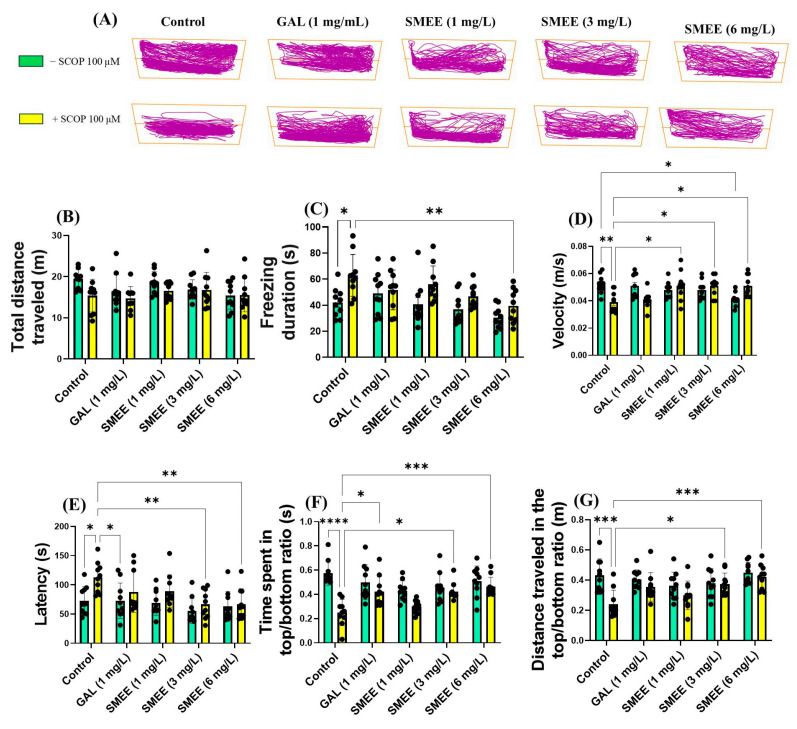
Effects of SMEE treatment (1, 3, and 6 mg/L) on native and scopolamine (SCOP, 100 µM)-treated zebrafish in the NTT. Galantamine (GAL, 1 mg/L) served as a positive control. (**A**) Representative swimming paths; (**B**) total distance traveled (m); (**C**) freezing duration (s); (**D**) velocity (m/s); (**E**) latency(s); (**F**) ratio of time spent in the top versus bottom zone (s); and (**G**) ratio of the distance traveled in the top versus bottom zone. Data are presented as mean values ± S.E.M. with *n* = 10 animals per group. Statistical analysis was performed using the Tukey HSD post hoc test: * *p* < 0.05, ** *p* < 0.001, *** *p* < 0.0001, and **** *p* < 0.00001.

To assess whether SMEE treatment attenuates the anxiogenic response induced by SCOP, the novel tank diving test (NTT) was conducted. Representative swimming trajectories (Figure 3A) showed that SCOP-treated zebrafish displayed a strong preference for the bottom zone of the tank, indicative of elevated anxiety-like behavior. In contrast, zebrafish treated with SMEE exhibited increased exploration of the top zone, suggesting an anxiolytic effect. Two-way ANOVA revealed a significant main effect of treatment on the freezing duration (F (1, 90) = 19.27, *p* < 0.0001) (Figure 3C) and velocity (F (1, 90) = 9.027, *p* < 0.001) (Figure 3D), indicating that SMEE administration significantly reduced anxiety-related freezing and improved locomotor activity, independent of SCOP exposure. No significant main effect was observed for the total distance traveled (F (1, 90) = 5.541, *p* = 0.0207) (Figure 3B), suggesting that SMEE treatment did not generally alter overall activity levels. For anxiety-specific behavior, two-way ANOVA indicated a significant main effect of treatment on latency (F (1, 90) = 11.56, *p* = 0.0010) (Figure 3E), time spent in the top/bottom ratio (F (1, 90) = 34.95, *p* < 0.0001) (Figure 3F), and distance traveled in the the top/bottom ratio (F (1, 90) = 14.78, *p* = 0.0002) (Figure 3G). Tukey HSD post hoc comparisons revealed that, in SCOP-treated zebrafish, SMEE significantly reduced the freezing duration (6 mg/L, *p* < 0.01) (Figure 3C) and increased the swimming speed at all concentrations tested (1, 3, and 6 mg/L, *p* < 0.05) (Figure 3D), relative to SCOP-only controls. Moreover, SMEE treatment improved exploratory behavior by decreasing latency to the top zone (3 and 6 mg/L, *p* < 0.01) (Figure 3E) and increasing both the time spent (3 mg/L, *p* < 0.05 and 6 mg/L, *p* < 0.001) (Figure 3F) and the distance traveled in the top zone (3 mg/L, *p* < 0.05 and 6 mg/L, *p* < 0.001) (Figure 3G). GAL (1 mg/L) was used as a reference drug in the NTT and significantly increased the time taken to explore the top zone (*p* < 0.05) (Figure 3F).

**Figure 4:**
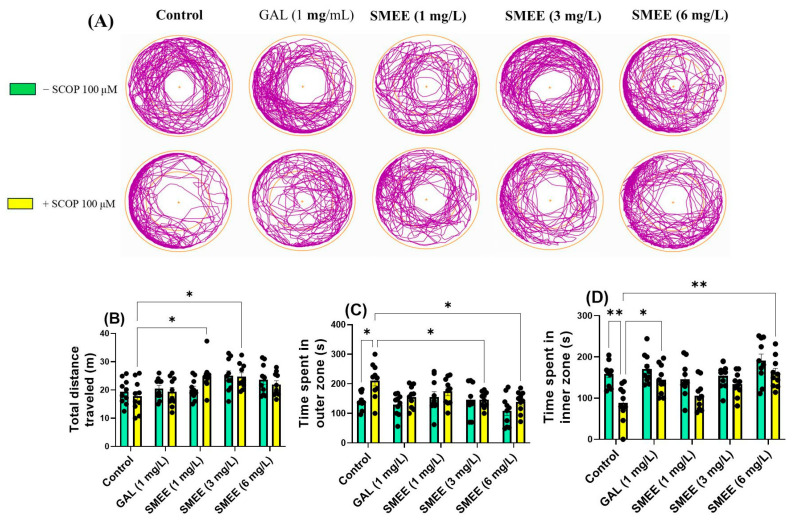
Effects of SMEE (1, 3, and 6 mg/L) on native and scopolamine (SCOP, 100 µM)-treated zebrafish in the NAT. Galantamine (GAL, 1 mg/L) served as a positive control. (**A**) Representative swimming trajectories; (**B**) total distance traveled (m); (**C**) time spent in outer zone (s); and (**D**) time spent in inner zone (s). Data are presented as mean values ± S.E.M. (*n* = 10 animals per group). Statistical analysis was performed using the Tukey HSD post hoc test: * *p* < 0.05 and ** *p* < 0.001.

To further assess anxiety-like behavior, the novel approach test (NAT) was employed. SCOP-treated zebrafish displayed increased thigmotaxis, spending more time exploring the outer zone of the tank, indicative of heightened anxiety-like behavior. SMEE treatment appeared to reduce this anxiety-related avoidance (Figure 4A). Two-way ANOVA revealed a significant main effect of treatment on the total distance traveled (F (4, 90) = 5.622, *p* < 0.05) (Figure 4B), time spent in the outer zone (F (1, 90) = 10.73, *p* = 0.0015) (Figure 4C), and time spent in the inner zone (F (1, 90) = 24.87, *p* < 0.0001) (Figure 4D). These results indicate that SMEE administration significantly influenced both locomotor activity and anxiety-like behavior across groups. No significant treatment × cohort interaction was detected, suggesting that the effect of SMEE on anxiety-like behavior was consistent regardless of SCOP exposure. A Tukey HSD post hoc test showed that acute SCOP and SMEE administration (1 and 3 mg/L) significantly increased locomotion and the total distance traveled (*p* < 0.05) (Figure 4B). In the SCOP-exposed groups, SMEE treatment at 3 and 6 mg/L significantly reduced the time spent in the outer zone (*p* < 0.05) (Figure 4C) and increased the time spent in the inner zone (6 mg/L, *p* < 0.001) (Figure 4D), indicating an anxiolytic-like effect. Also, GAL (1 mg/L), used as a reference drug in NAT, caused a significant increase in the time spent exploring the inner zone (*p* < 0.05) (Figure 4D).

**Figure 5:**
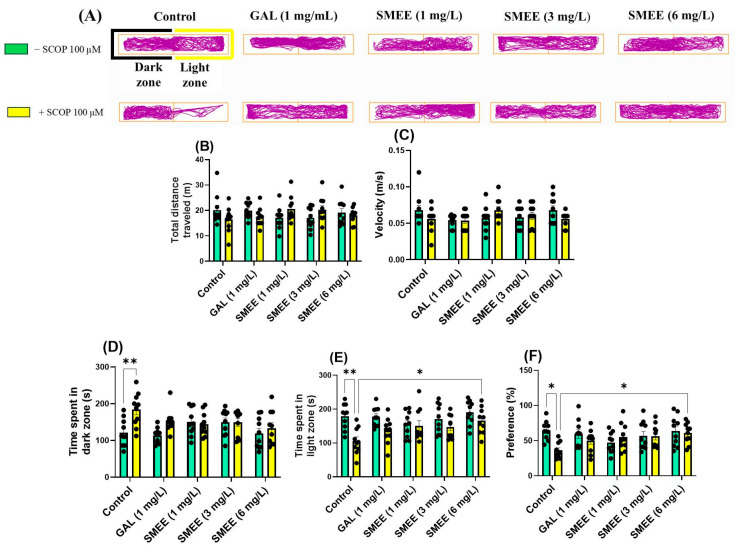
Effects of SMEE (1, 3, and 6 mg/L) on native and scopolamine (SCOP, 100 µM)-treated zebrafish in the LDT. Galantamine (GAL, 1 mg/L) served as a positive control. (**A**) Representative swimming paths; (**B**) total distance traveled (m); (**C**) velocity (m/s); (**D**) time spent in light zone (s); (**E**) time spent in dark zone (s); and (**F**) preference (%). Data are presented as mean values ± S.E.M. (*n* = 10 animals per group). Statistical significance was determined using the Tukey HSD post hoc test: * *p* < 0.05 and ** *p* < 0.001.

To further evaluate anxiety-like responses, the light–dark test (LDT) was employed. SCOP-treated zebrafish exhibited increased avoidance of the light zone, spending more time in the dark zone, a behavior commonly associated with anxiety. In contrast, zebrafish treated with SMEE showed increased exploration of the light zone, suggestive of anxiolytic activity (Figure 5A). Two-way ANOVA revealed a significant main effect of treatment on time spent in the dark zone (F (1, 90) = 8.840, *p* = 0.0038) (Figure 5D), time spent in the light zone (F (1, 90) = 19.11, *p* < 0.0001) (Figure 5E), and preference for the light zone (F (1, 90) = 3.837, *p* = 0.0532) (Figure 5F). No significant main effects or interactions were found for the total distance traveled (F (1, 90) = 2.663, *p* = 0.0376) (Figure 5B) or velocity (F (1, 90) = 2.154, *p* = 0.0806) (Figure 5C), suggesting that SMEE treatment did not alter general locomotor activity. No significant treatment × cohort interaction was detected for any anxiety-related measure, indicating consistent effects of SMEE across SCOP and non-SCOP groups. The Tukey HSD post hoc test revealed that SCOP significantly increased time spent in the dark zone compared to controls (*p* < 0.01) (Figure 5D). In SCOP-treated zebrafish, SMEE at 6 mg/L significantly increased time spent in the light zone (*p* < 0.05) (Figure 5E) and light zone preference (*p* < 0.05) (Figure 5F), indicating an anxiolytic-like effect. The doses of SMEE (1 and 3 mg/L) showed similar trends, though the differences did not reach statistical significance. These findings suggest that chronic SMEE administration counteracts SCOP-induced anxiety by promoting light zone exploration in the LDT, a behavior associated with reduced anxiety levels in zebrafish.

**Figure 6:**
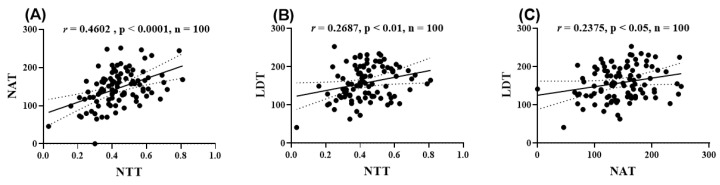
Correlation of zebrafish residence time in the top/bottom ratio (NTT), light (LDT), and inner (NAT) zones. (**A**) Residence time in the top/bottom ratio (NTT) relative to duration of inner zone exploration (NAT); (**B**) duration of the top/bottom ratio (NTT) zone exploration relative to residence time in the light zone (LDT); (**C**) duration of light zone exploration (LDT) relative to duration of inner zone exploration (NAT) (*n* = 100) (Pearson correlations).

Figure 6 illustrates the correlations between the behavioral parameters in control, SCOP-treated, and SCOP + SMEE-treated zebrafish (1, 3, and 6 mg/L). A strong positive correlation was observed between the time spent in the top zone during the NTT and the time spent in the inner zone during the NAT (r = 0.4602; *p* < 0.0001; *n* = 100) (Figure 6A). Simultaneously, a significant positive correlation between the time spent in the top zone (NTT) and the time spent in the light zone (LDT) (r = 0.2687; *p* < 0.01; *n* = 100) was noted (Figure 6B). Furthermore, a positive correlation was also identified between the time spent in the light zone (LDT) and the time spent in the dark zone (NAT) (r = 2375; *p* < 0.01; *n* = 100) (Figure 6C).

### 2.6. Effects on Spatial Memory in Y-Maze

**Figure 7:**
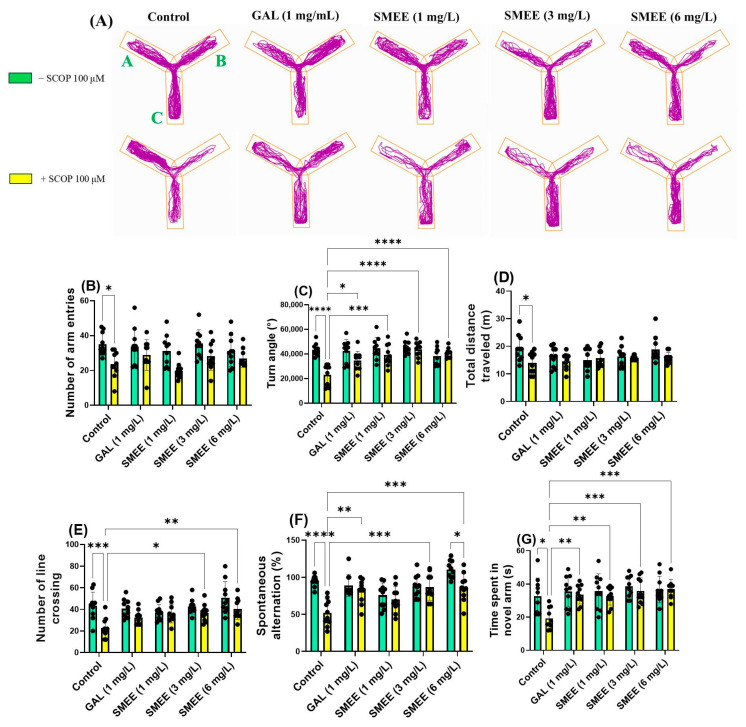
Effects of SMEE (1, 3, and 6 mg/L) on native and scopolamine (SCOP, 100 µM)-treated zebrafish in the Y-maze test. Galantamine (GAL, 1 mg/L) served as a positive control. For the Y-maze: A—start arm; B—novel arm; C—other arm. (**A**) Representative swimming trajectories; (**B**) number of arm entries; (**C**) turn angle (°); (**D**) total distance traveled (m); (**E**) number of line crossing; (**F**) spontaneous alternation (%); and (**G**) time spent in novel arm (s). Data are presented as mean values ± S.E.M. (*n* = 10 animals per group). Statistical significance was determined using the Tukey HSD post hoc test: * *p* < 0.05, ** *p* < 0.001, *** *p* < 0.0001, and **** *p* < 0.00001.

The Y-maze test assessed spatial memory and exploratory behavior by measuring zebrafish responses to a novel arm. SCOP-treated fish exhibited reduced activity in the novel arm, indicating impaired spatial memory, while SMEE-treated fish demonstrated improved exploration of the novel arm, consistent with memory enhancement (Figure 7A). Two-way ANOVA revealed a significant main effect of treatment on the number of arm entries (F (1, 90) = 24.88, *p* < 0.0001) (Figure 7B), turn angle (F (1, 90) = 20.89, *p* < 0.0001) (Figure 7C), and total distance traveled (F (1, 90) = 8.049, *p* = 0.0056) (Figure 7D), indicating that SMEE improved general exploratory behavior and locomotion. In terms of spatial memory, a significant main effect of treatment was also found for the number of line crossings (F (1, 90) = 23.40, *p* < 0.0001) (Figure 7E), spontaneous alternation (F (1, 90) = 27.79, *p* < 0.0001) (Figure 7F), and time spent in the novel arm (F (1, 90) = 6.630, *p* = 0.0117) (Figure 7G), suggesting treatment-related memory improvement. No significant treatment × cohort interaction was detected, indicating that SMEE had a consistent effect on these parameters in both SCOP and non-SCOP groups. The Tukey HSD post hoc test confirmed that SCOP exposure significantly reduced the number of arm entries (*p* < 0.05) (Figure 7B), the turn angle (*p* < 0.0001) (Figure 7C), the total distance traveled (*p* < 0.05) (Figure 7D), the number of line crossings (*p* < 0.001) (Figure 7E), the spontaneous alternation (*p* < 0.0001) (Figure 7F), and the time spent in the novel arm (*p* < 0.001) (Figure 7G) compared to the control group. In contrast, SMEE treatment reversed these effects in a dose-dependent manner. SMEE at 3 and 6 mg/L significantly increased the number of line crossings (*p* < 0.05 and *p* < 0.01, respectively) (Figure 7E), spontaneous alternation percentage (*p* < 0.001) (Figure 7F), and time spent in the novel arm (1 mg/L, *p* < 0.01; 3 and 6 mg/L, *p* < 0.001) (Figure 7G) compared to the SCOP-only group. The reference drug GAL (1 mg/L) also significantly improved these parameters. These findings suggest that SMEE mitigates SCOP-induced impairments in spatial working memory and enhances exploratory activity, supporting its cognitive-enhancing potential.

### 2.7. Effects on Recognition Memory in NOR

**Figure 8:**
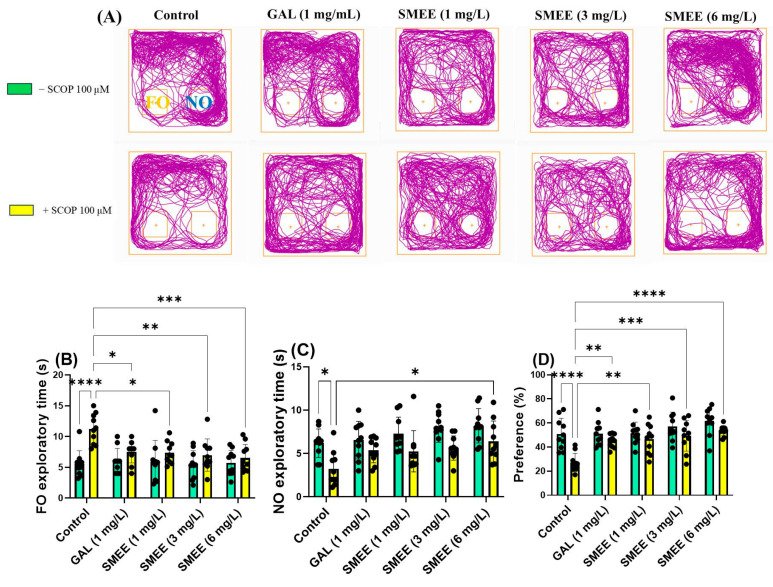
Effects of SMEE (1, 3, and 6 mg/L) on native and scopolamine (SCOP, 100 µM)-treated zebrafish in the NOR. Galantamine (GAL, 1 mg/L) served as a positive control. (**A**) Representative swimming trajectories; (**B**) familiar object (FO) exploratory time (s); (**C**) novel object (NO) exploratory time (s); and (**D**) preference (%). Data are presented as mean values ± S.E.M. (*n* = 10 animals per group). Statistical significance was determined using the Tukey HSD post hoc test: * *p* < 0.05, ** *p* < 0.01, *** *p* < 0.001, and **** *p* < 0.0001.

The novel object recognition test (NOR) was used to evaluate recognition memory by measuring the time zebrafish spent exploring a novel object (NO) versus a familiar one (FO). SCOP-treated fish showed a reduced preference for the novel object, indicating recognition memory impairment. In contrast, SMEE-treated fish demonstrated improved NO exploration, consistent with memory enhancement (Figure 8A). Two-way ANOVA revealed a significant main effect of treatment on the time spent exploring the FO (F (1, 90) = 22.27, *p* < 0.0001) (Figure 8B), time spent exploring the NO (F (1, 90) = 28.84, *p* < 0.0001) (Figure 8C), and preference for the NO (F (1, 90) = 24.17, *p* < 0.0001) (Figure 8D), indicating that treatment significantly influenced recognition memory performance. No significant treatment × cohort interaction was found, suggesting that the effect of SMEE on recognition memory was consistent across both SCOP-treated and untreated groups. The Tukey HSD post hoc test confirmed that SCOP administration significantly impaired recognition memory by increasing the time spent on the FO (*p* < 0.0001) (Figure 8B) and reducing both NO exploration (*p* < 0.05) (Figure 8C) and preference (*p* < 0.05) (Figure 8D) compared to controls. In contrast, SMEE treatment produced a dose-dependent improvement in recognition memory. SMEE at 1, 3, and 6 mg/L significantly reduced FO exploration (*p* < 0.05, *p* < 0.01, and *p* < 0.001, respectively) (Figure 8B). SMEE at 6 mg/L significantly increased the time spent on the NO (*p* < 0.05) (Figure 8C), and all doses significantly increased the preference for the NO (1 mg/L, *p* < 0.01; 3 mg/L, *p* < 0.001; 6 mg/L, *p* < 0.0001) versus SCOP-only fish (Figure 8D). The reference drug GAL (1 mg/L) also significantly improved NO preference (*p* < 0.01) (Figure 8D).

### 2.8. Effects on the Brain AChE Activity

**Figure 9:**
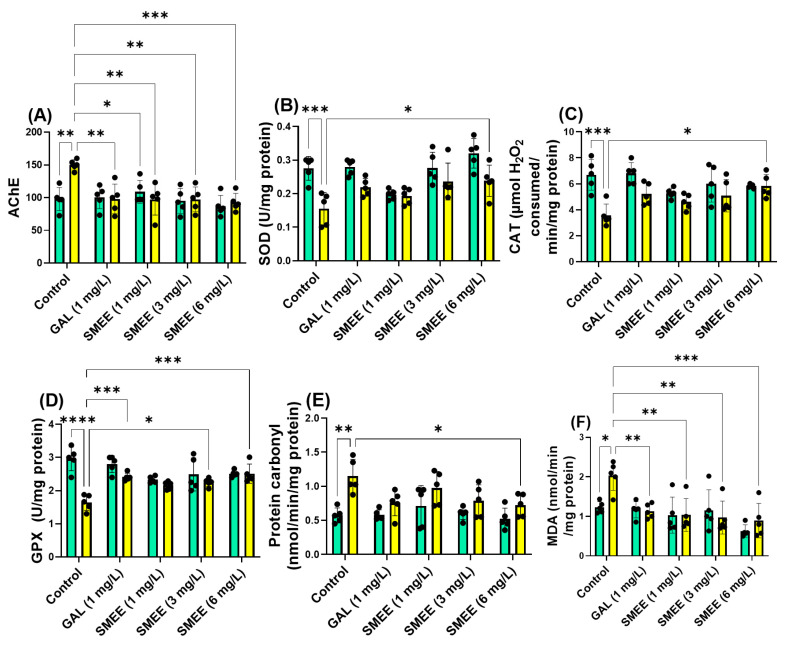
The impact of SMEE treatment (1, 3, and 6 mg/L) on native and scopolamine (SCOP, 100 µM)-treated zebrafish on the following biochemical parameters: (**A**) acetylcholinesterase (AChE) activity; (**B**) superoxide dismutase (SOD) specific activity; (**C**) catalase (CAT) specific activity; (**D**) glutathione peroxidase (GPX) specific activity; (**E**) carbonylated protein levels; and (**F**) malondialdehyde (MDA) content. Data are presented as mean values ± S.E.M. (*n* = 5 animals per group). Statistical significance based on the Tukey HSD post hoc test: * *p* < 0.05, ** *p* < 0.001, *** *p* < 0.0001, and **** *p* < 0.00001. Green bar: − SCOP 100 μM and yellow bar: + SCOP 100 μM.

Acetylcholinesterase (AChE) activity was measured in the zebrafish brain to assess cholinergic system modulation following SMEE treatment and SCOP exposure (Figure 9A). SCOP treatment significantly increased AChE activity compared to the control group, reflecting cholinergic disruption and mimicking a key feature of AD-like pathology. In contrast, SMEE treatment decreased AChE activity, suggesting a potential mechanism for cognitive protection. Two-way ANOVA revealed a significant main effect of treatment on AChE activity (F (1, 40) = 4.786, *p* = 0.0030) (Figure 9A), indicating that SMEE significantly influenced cholinergic enzyme activity across groups. No significant treatment × cohort interaction was found, suggesting that the inhibitory effect of SMEE on AChE activity occurred both in the presence and absence of SCOP-induced neurotoxicity. The Tukey HSD post hoc test confirmed that SCOP exposure significantly increased AChE activity compared to controls (*p* < 0.01). SMEE treatment at 1 and 3 mg/L significantly reduced AChE activity compared to the SCOP-only group (*p* < 0.01), while the 6 mg/L dose resulted in an even stronger inhibitory effect (*p* < 0.001). The reference drug GAL (1 mg/L) also significantly decreased AChE activity relative to SCOP (*p* < 0.01). These results suggest that SMEE reverses SCOP-induced cholinergic dysfunction by inhibiting AChE activity, which may underlie its memory-enhancing and neuroprotective effects in the zebrafish model.

### 2.9. Effects on Brain Oxidative Status

To assess the antioxidant potential of SMEE and its ability to reverse oxidative stress induced by SCOP, the activities of key brain antioxidant enzymes and oxidative damage markers were analyzed (Figure 9B–F). SCOP exposure significantly impaired the brain’s antioxidant defense system, as evidenced by reduced enzymatic activity (superoxide dismutase, SOD; catalase, CAT; glutathione peroxidase, GPX) and elevated oxidative stress markers (protein carbonyl and malondialdehyde, MDA). SMEE treatment counteracted these effects, suggesting a neuroprotective role mediated by redox modulation. Two-way ANOVA revealed significant main effects of treatment on the SOD activity (F (1, 40) = 31.50, *p* < 0.0001) (Figure 9B), CAT (F (1, 40) = 23.23, *p* < 0.0001) (Figure 9C), GPX (F (1, 40) = 34.98, *p* < 0.0001) (Figure 9D), carbonyl protein concentration (F (1, 40) = 25.89, *p* < 0.0001) (Figure 9E), and malondialdehyde (MDA) levels (F (1, 40) = 7.856, *p* < 0.0001) (Figure 9F). No significant treatment × cohort interactions were detected, suggesting that the effects of SMEE on oxidative stress parameters were consistent regardless of SCOP exposure. The Tukey HSD post hoc test showed that SCOP significantly reduced SOD (*p* < 0.001) (Figure 9B), CAT (*p* < 0.001) (Figure 9C), GPX (*p* < 0.0001) (Figure 9D) and elevated carbonylated protein (*p* < 0.01) (Figure 9E) and MDA levels (*p* < 0.05) (Figure 9F) compared to the control group. In contrast, SMEE administration at 6 mg/L significantly increased SOD (*p* < 0.05) (Figure 9B), CAT (*p* < 0.05) (Figure 9C), and GPX activity (3 mg/L, *p* < 0.05; 6 mg/L, *p* < 0.001) (Figure 9D), while reducing carbonylated proteins (6 mg/L, *p* < 0.05) (Figure 9E) and MDA levels (1 and 3 mg/L, *p* < 0.01; 6 mg/L, *p* < 0.001). These improvements were comparable to those observed with the reference drug GAL. These findings indicate that SMEE enhances the antioxidant defense system and reduces oxidative damage in the brain, likely contributing to its neuroprotective effects in SCOP-induced cognitive impairment.

### 2.10. Pearson Correlations Between Behavioral and Biochemical Variables

**Figure 10:**
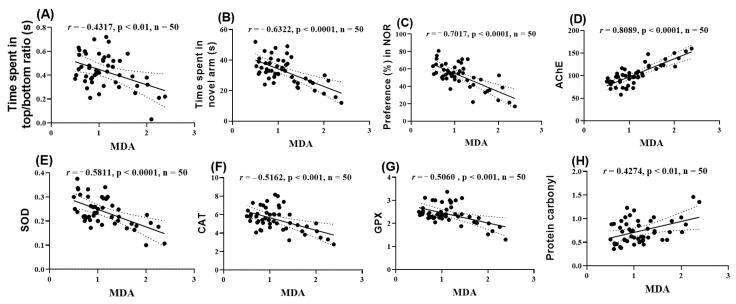
Correlation analyses between behavioral and biochemical parameters (Pearson correlation). Data presented are the (**A**) time spent in the top/bottom zone of the NTT compared to MDA levels (*n* = 50, r = −0.4317, *p* < 0.01); (**B**) time spent in the novel arm of the Y-maze compared to MDA (*n* = 50, r = −0.6322, *p* < 0.0001); (**C**) percentage preference in the NOR compared to MDA (*n* = 50, r = −0.7017, *p* < 0.0001); (**D**) AChE activity compared to MDA (*n* = 50, r = 0.8089, *p* < 0.0001); (**E**) SOD activity compared to MDA (*n* = 50, r = −0.5811, *p* < 0.0001); (**F**) CAT activity compared to MDA (*n* = 50, r = −0.5162, *p* < 0.001); (**G**) GPX activity compared to MDA (*n* = 50, r = −0.5060, *p* < 0.001); and (**H**) carbonylated protein levels compared to MDA (*n* = 50, r = 0.4274, *p* < 0.01).

The Pearson correlation coefficient (r) was used to assess the relationships between behavioral parameters, enzymatic activities, and lipid peroxidation. The analyzed parameters included the time spent in the top and bottom zones of the tank, the exploration time of the novel arm in the Y-maze, the percentage of preference, as well as the values for AChE, SOD, CAT, GPX, and carbonylated proteins reported to MDA (Figure 10). Thus, it was found that the time spent in the top/bottom zones (Figure 10A), the time exploring the novel arm (Figure 10B), the percentage of preference (Figure 10C), and the activities of SOD (Figure 10E), CAT (Figure 10F), and GPX (Figure 10G) showed significant negative correlations with MDA (r = −0.4317, −0.6322, −0.7017, −0.5811, −0.5162, and −0.5060, respectively). In contrast, the activity of AChE (Figure 10D) and the level of carbonylated proteins (Figure 10H) showed significant positive correlations with MDA (r = 0.8089 and 0.4274, respectively).

## 3. Discussion

This study was undertaken to investigate, for the first time, the neuroprotective and anxiolytic potential of SMEE in a zebrafish model of SCOP-induced cognitive impairment and anxiety-like behavior. Although previous reports have described antioxidant and anti-inflammatory properties of *S. macrocarpon*, its effects on central nervous system function, particularly in validated in vivo models of neurodegeneration, remain unexplored. Our findings demonstrate that SMEE significantly improved cognitive performance (Y-maze, NOR tests) and reduced anxiety-like behavior (NTT, NAT, LDT) in zebrafish exposed to SCOP. These behavioral effects were supported by biochemical evidence showing reduced brain AChE activity and enhanced antioxidant enzyme activity (SOD, CAT, GPX), along with decreased levels of lipid peroxidation (MDA) and protein oxidation (carbonylated proteins) markers.

In this investigation, the polyphenolic compounds from the SMEE were analyzed using the HPLC technique. The results obtained highlighted that the main components of the extract are chlorogenic acid and rutin. Previous studies on the polyphenolic compounds in the SMEE have demonstrated the presence of compounds with significant antioxidant potential. In the study by Salawu et al. [19], HPLC analyses identified derivatives of chlorogenic acid, rutin, and kaempferol-3-rutinoside, which are recognized for their antioxidant properties and potential contribution to protecting against oxidative stress.

Immediately after the HPLC analysis, an in silico pharmacokinetic evaluation was performed using the ADMET model for the two identified bioactive compounds. To complement our in vivo findings, we made in silico ADMET and PASS predictions, which offered insight into the pharmacokinetic properties and potential targets of SMEE constituents, although some predicted toxicities, such as for rutin and chlorogenic acid, appeared inconsistent with their observed neuroprotective effects in the zebrafish model.

The in silico ADMET and PASS predictions provided useful preliminary insights into the pharmacokinetic and toxicological properties of phytoconstituents identified in SMEE. Interestingly, some compounds, such as rutin and chlorogenic acid, showed predicted neurotoxicity, despite their well-established neuroprotective and antioxidant effects reported in vivo and supported by the current findings. This discrepancy highlights a common limitation of predictive tools, which may not fully capture the complexity of pharmacodynamics, including bioavailability, metabolic transformation, tissue distribution, or synergistic effects with other constituents. Therefore, while useful for early screening, such predictions should be interpreted with caution and always validated with experimental data, as carried out in this study.

The ADMET analysis suggests that SCOP and GAL exhibit higher oral bioavailability, a more extensive systemic distribution, and an active hepatic metabolism profile, but also a higher toxicological risk, particularly at the hepatic and cardiac levels. On the other hand, chlorogenic acid and rutin have lower intestinal absorption, slower elimination, and a different toxicological risk, especially regarding the hepatotoxic and genotoxic effects of rutin. These findings are essential for assessing the therapeutic potential and safety of the analyzed compounds, supporting their pharmacological use in low concentrations, considering the principle of neurohormesis [6]. In silico ADMET predictions suggest that key phytoconstituents such as chlorogenic acid and rutin possess good BBB permeability, supporting the likelihood that active compounds reached brain tissue to exert central effects. The probable mechanism of action involves a combination of cholinesterase inhibition, direct free radical scavenging, and modulation of endogenous antioxidant systems, leading to reduced oxidative damage and preservation of cholinergic neurotransmission—all of which are key targets in neurodegenerative conditions like AD.

Chlorogenic acid is a polyphenol with strong antioxidant, anti-inflammatory, antimicrobial, antidiabetic, and antitumor effects. Additionally, it exhibits hepatoprotective, nephroprotective, and neuroprotective properties. Its benefits are mediated through NF-kB pathway inhibition (reducing inflammation), Nrf2 pathway activation (reducing oxidative stress), and AMPK stimulation. These mechanisms support its therapeutic potential in various diseases [20,21,22]. Rutin is a polyphenolic flavonoid with multiple pharmacological effects, including antioxidant and anti-inflammatory [23], antimicrobial, antidiabetic, and antitumor activity [24]. Additionally, it exhibits hepatoprotective, nephroprotective, and neuroprotective properties. Pharmacokinetically, rutin has limited intestinal absorption and low bioavailability, being primarily metabolized in the intestine and liver, with elimination occurring through urine and feces. Toxicologically, rutin is considered safe at therapeutic doses, but high doses may cause minor adverse effects such as gastrointestinal discomfort or allergic reactions. Its pharmacokinetic limitations necessitate optimization strategies to enhance its clinical efficacy [25].

Subsequently, three concentrations of SMEE (1, 3, and 6 mg/L) were chronically administered to zebrafish treated with SCOP to investigate the potential for ameliorating behavioral deficits induced by SCOP. This study focused on assessing behavioral effects through well-established in vivo tasks, such as anxiety tests: NTT, NAT, and LDT, and memory tests: Y-maze and NOR. The results obtained indicate that SMEE treatment significantly reduced SCOP-induced anxiety behavior in the NTT, NAT, and LDT tests and counteracted the memory deficits revealed in the Y-maze and NOR tasks. These findings are consistent with both the literature and the results of previous studies conducted by our group, which demonstrated that exposure to SCOP causes variable behavioral changes, influenced mainly by the dose administered and the duration of exposure [6,26]. For example, in our previous investigations, exposure for 30 min to a concentration of 100 µM SCOP generated anxiogenic effects and negatively affected spatial and recognition memory [6]. In addition, studies by other research groups have demonstrated that SCOP administration can induce cognitive impairment and anxiety symptoms, similar to those found in AD [27]. However, it is noted that, at high concentrations (800 µM), SCOP hydrobromide produces anxiolytic effects in zebrafish [28].

This phenomenon can be explained by the fact that, unlike SCOP, SCOP hydrobromide does not cross the BBB due to the presence of its quaternary ammonium salt fragment, which prevents the occurrence of direct adverse effects on the CNS [29]. The effect of SMEE on the specific activity of AChE was analyzed in the brains of zebrafish exposed to SCOP, considering the crucial role of this enzyme in the regulation of cholinergic neurotransmission. The results obtained indicated that SMEE administration caused a significant inhibition of AChE activity, which was considerably amplified following SCOP exposure. This suppression of enzymatic activity suggests a potential neuroprotective mechanism of SMEE, through which it may contribute to the maintenance of cholinergic homeostasis and to the reduction in cognitive dysfunctions associated with AChE hyperactivity. Thus, SMEE could have a beneficial effect in preventing or ameliorating cognitive decline induced by disturbances of the cholinergic system. A study on the effects of aqueous extract of *S. macrocarpon* on cholinesterase activity in alloxan-induced diabetes showed a significant increase in AChE and BChE in diabetic rats. After 14 days of treatment, the extract significantly reduced AChE activity, especially at doses of 24.9 and 49.8 mg/kg, which brought the levels to values comparable to those of normoglycemic controls. In addition, the dose of 12.45 mg/kg was more effective than metformin (5 mg/kg). In contrast, BChE did not show significant changes between groups, suggesting a selective effect on AChE [17]. A previous study by Ogunsuyi et al. [30] revealed in the leaves of *S. macrocarpon* the presence of phenolic compounds and alkaloids with inhibitory activity on AChE, suggesting a neuroprotective and anti-inflammatory potential. Molecular docking analyses and MD simulations indicated that the flavonoids luteolin–retinoid (LR), especially LR4 and LR5, act as AChE inhibitors through double binding, interacting with both the catalytic triad and the peripheral anionic site. Similarly, Idowu et al. [31] showed that *S. macrocarpon* induces significant inhibition of AChE, suggesting a neuroprotective potential. The ethyl acetate fraction showed the highest activity, and phytochemical analysis identified compounds with a high affinity for AChE, comparable to donepezil. Computational studies confirmed the stability of the interaction, supporting the use of *S. macrocarpon* as a source of natural AChE inhibitors.

The antioxidant effect of SMEE was analyzed in the experimental zebrafish model exposed to SCOP by evaluating the specific activity of the main antioxidant enzymes and the degree of lipid peroxidation and protein oxidation. The results obtained demonstrate that SMEE possesses significant antioxidant properties, contributing to the reduction in oxidative stress induced by SCOP. This beneficial effect is highlighted by the increase in the activities of SOD, CAT, and GPX, key enzymes involved in the neutralization of reactive oxygen species. In parallel, SMEE caused a significant decrease in the levels of MDA, a marker of lipid peroxidation, and carbonylated proteins, indicators of oxidative degradation of proteins [6]. Recently, Okesola et al. [17], Etuk et al. [32], and Osukoya et al. [33] have highlighted the antioxidant potential of ethanolic and aqueous extracts of *S. macrocarpon* to ameliorate alloxan-induced oxidative stress in rodents by regulating the levels of oxidative stress markers (SOD, CAT, GPX, and GSH).

Therefore, our results suggest that SMEE may exert neuroprotective and anxiolytic effects, possibly by modulating the cholinergic system, reducing oxidative stress, or through other mechanisms of neurotransmission regulation. Thus, SMEE intervention proves to be promising in counteracting the cognitive and behavioral deficits induced by SCOP, highlighting its therapeutic potential in the management of disorders associated with cognitive dysfunction and anxiety. To the best of our knowledge, this is the first study to demonstrate the neuroprotective and anxiolytic effects of SMEE in a SCOP-induced zebrafish model of cognitive impairment. Previous research on *S. macrocarpon* primarily focused on its antioxidant and metabolic benefits [17,18], but its potential role in modulating cholinergic dysfunction, oxidative stress, and behavioral deficits associated with dementia-related pathologies had not previously been investigated. Our findings thus provide novel evidence supporting the therapeutic potential of *S. macrocarpon* in neurodegenerative disease models.

Although the results obtained suggest a promising therapeutic potential of SMEE in improving the cognitive and behavioral deficits induced by SCOP in zebrafish, certain limitations must be considered. First, the absence of mammalian validation limits the translational potential of our findings, although zebrafish remain a valuable high-throughput model for early-stage neurobehavioral screening. Second, as with most herbal-based extracts, the phytochemical composition may vary between batches, and future standardization is needed to improve reproducibility. Third, while DMSO was used as a vehicle at a concentration of 1%—a range generally considered non-toxic in zebrafish—it cannot be entirely excluded that even low levels of DMSO may influence behavior. Finally, the current study did not perform sex-specific analysis; fish were randomly assigned to treatment groups without sex identification. As sex differences in zebrafish behavior and pharmacodynamics have been reported, future studies should incorporate sex-balanced or stratified designs to clarify whether the observed effects are sex-dependent.

To confirm and extend the results obtained, future studies should include behavioral and biochemical evaluations in rodent animal models to validate the efficacy of SMEE under physiological conditions closer to those of humans. Also, the detailed characterization of the active compounds in SMEE and the elucidation of their molecular mechanisms of action, including through molecular techniques (proteomics, metabolomics), could provide essential information for the development of therapies based on natural extracts. Although the observed behavioral and biochemical effects suggest central activity, no direct biodistribution or imaging analysis was performed to confirm the passage of active compounds across the BBB or their localization in specific brain regions. Future studies using pharmacokinetic or neuroimaging techniques are needed to validate brain penetration and regional targeting of the extract’s constituents. Finally, preliminary clinical studies could provide further evidence on the efficacy and safety of SMEE in improving cognitive and anxiolytic disorders in humans, paving the way for possible medical applications.

## 4. Materials and Methods

### 4.1. Plant Material and Extraction

*S. macrocarpon* was purchased from the local market in July 2021, in Oyo, Nigeria. The plant was identified by Prof. Omokafe Ugbogu and authenticated at the Forest Herbarium Ibadan (FHI), with herbarium number FHI 113641. The voucher specimen of the plant was deposited in the mentioned herbarium.

Leaves were detached from the stem, and leaf samples were frozen and ground into powder. Powdered samples were macerated in 96% ethanol for 72 h and filtered. Maceration was repeated until the green color of the filtrate faded. The extract was filtered and concentrated by using a rotary evaporator, resulting in the SMEE.

### 4.2. High-Performance Liquid Chromatograph (HPLC-PDA)

For quantitative determination of the phenolic compounds, 5 mg/mL of SMEE was dissolved in 50% acetonitrile (ACN) and filtered through a 0.20 µm cellulose acetate membrane filter before the analysis. Twenty-three standards (rutin, resveratrol, chlorogenic, caffeic, and *p*-coumaric acids, catechin hydrate, hesperetin, myricetin, morin, apigenin, quercetin, kaempferol, amentoflavone, rosmarinic acid, gallic acid, vanillic acid, syringic acid, ferulic acid, tyrosol, oleuropein, ellagic acid, naringin, and epigallocatechin gallate) comprising phenolic acids and flavonoids were analyzed for this study. Phenolic acid standards were dissolved in 25% ACN, while flavonoids were dissolved in 50% ACN. For the calibration curve, peak areas were plotted against five concentrations of the standards (1, 10, 25, 100, and 200 ppm). Phenolic and flavonoid compounds were determined and quantified by reverse phase HPLC (Agilent Technologies, Santa Clara, CA, USA) on an ACE 5 (150 × 4.6 mm, 5 μm, 25 °C) C18 column following the method described by Ekin et al. [34]. The mobile phase system contained 0.1% formic acid (FA) in acetonitrile 80% (solvent A) and 0.1% FA in H_2_O (solvent B). The gradient system was set as follows: from 5% A to 15% A with a 0.8 mL/min flow rate for 10 min, 15% A with a flow rate from 0.8 to 0.6 mL/min for 5 min, 15% A with a 0.6 mL/min flow for 2 min, from 15% to 20% A with 0.8 mL/min flow for 5 min, from 20% to 30% A with 0.8 mL/min flow for 4 min, from 30% to 100% A with 0.8 mL/min flow for 8 min, 100% A at 1 mL/min flow for 3 min, and returned to the initial conditions within 5 min. We injected 20 μL of the samples. The detection wavelengths used were 260, 280, 320, and 350 nm.

### 4.3. Computational Estimation of the Pharmacokinetic Profile

The pharmacokinetic properties of the compounds quantified in SMEE extract, along with controls, i.e., SCOP and GAL, were analyzed using pKCSM [35] and ADMETlab 3.0 [36], as previously described [6,26]. The platforms were accessed on 20 February 2025. Parameters assessed included water solubility, Caco-2 permeability, intestinal absorption, P-glycoprotein interactions, skin permeability, volume of distribution (VDss), BBB penetration, central nervous system (CNS) permeability, CYP enzyme interactions, elimination, and toxicity (AMES, oral toxicity, hepatotoxicity, neurotoxicity, immunotoxicity, skin sensitization). Data were standardized and converted into binary categories for comparison.

### 4.4. Prediction of Biological Activity and Protein Target Identification

PASS [37] was used to predict the biological activity and off-target effects of the compounds, with results expressed as the probability of activity (Pa) and inactivity (Pi), where Pa > 0.5 indicated likely activity. The analyses provided insights into the pharmacological potential of the compounds. The platform was accessed on 20 February 2025.

### 4.5. Study Design and Animal Care

In this study, 100 adult wild zebrafish (5–8 months old, short-finned) were tested, with a male–female ratio of 1:1. The average body length of the fish ranged from 3 to 4 cm. All zebrafish used in this study were obtained from the European Zebrafish Resource Center at the Institute of Toxicology and Genetics in Germany and were placed in quarantine for two weeks upon arrival. During the quarantine period, the fish were housed in a 70 L glass tank with disinfected tap water, which was changed every two days. Water temperature was maintained at 27 ± 1 °C, and water parameters were monitored daily to ensure optimal conditions: pH 7–7.5, dissolved oxygen 8 ± 1 mg/L, conductivity 1400–1500 µS/cm, and ammonia/nitrite levels below 0.001 mg/L. The light cycle was set at 14 h light and 10 h dark. The fish were fed ad libitum with Norwin Norvital flakes (Norwin, Gadstrup, Denmark), in an automatic mode, three times a day (7 a.m., 2 p.m., and 7 p.m.), all food being consumed within 10 min.

**Figure 11:**
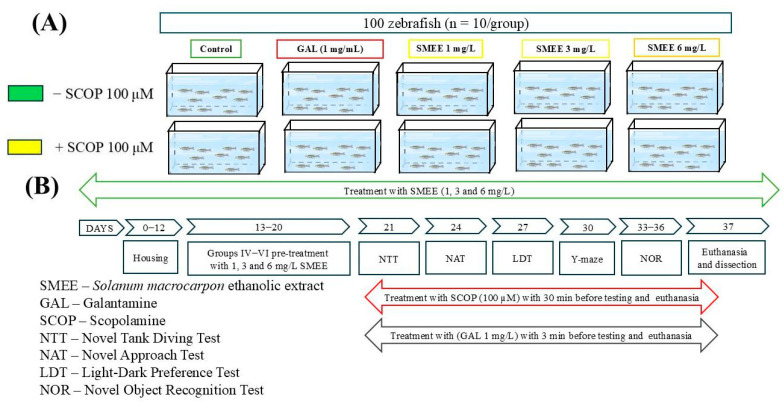
Schematic diagram of experimental design adopted in this study. (**A**) Experimental groups; (**B**) behavioral and biochemical tests.

Zebrafish were randomly assigned to two cohorts (±SCOP) of five experimental groups, each consisting of 10 fish equally distributed by size and sex (Figure 11A). The groups were as follows: (I) control; (II) GAL (1 mg/L), positive control for both behavioral and biochemical tests; (III–V) three treatment groups with ethanol extract fraction of SMEE (1, 3, and 6 mg/L); (VI) scopolamine (SCOP, 100 μM); (VII) SCOP (100 μM) + GAL (1 mg/L); and (VIII–X) SCOP (100 μM) + SMEE (1, 3, and 6 mg/L). GAL treatment was administered acutely for 3 min before testing in group II. SMEE was prepared in a 1% dimethyl sulfoxide (DMSO) solution and administered by immersion in water at 1, 3, and 6 mg/L for groups III–V and VIII–X, based on previous studies [15]. The zebrafish dementia model was induced in groups VI–X with SCOP (100 μM) for 30 min before behavioral tests and euthanasia, as described previously [6]. Group VII also received GAL (1 mg/L) for 3 min after SCOP treatment prior to testing (Figure 11B). Also, we confirmed that *n* = 10 fish/group was appropriate using InVivoStat, an R-based statistical package [38]. Based on a significance level of 0.05, the power to detect a 20% biologically relevant change was 97%. The data analysis and presentation, as well as this study’s experimental design, all followed the ARRIVE guidelines [39] for planning and organizing animal testing and research, respectively.

### 4.6. Behavioral Tasks

Zebrafish swimming behavior was recorded during in vivo assays using a Logitech C922 Pro HD Stream camera (Lausanne, Switzerland) and subsequently analyzed with ANY-maze software, version 7.48 (Stoelting Co., Wood Dale, IL, USA).

#### 4.6.1. Novel Tank Diving Test (NTT)

In the NTT, zebrafish exhibited a significant response to novel, anxiety-inducing stimuli. The protocol followed methods previously described by Cachat et al. [40]. The experiment was conducted in a trapezoidal tank containing 1.5 L of water, with dimensions of 23.9 cm at the base, 28.9 cm at the top, and 15.1 cm in height, with a diagonal side of 15.9 cm and a width of 7.4 cm at the top and 6.1 cm at the bottom. Each fish was placed individually in the tank, and its behavior was recorded for 6 min using a webcam. The tank was virtually divided into two zones: top and bottom. The water temperature was maintained at 27 ± 1 °C, and the water was changed between experimental groups. Anxious behaviors and locomotor activity were assessed according to the measurements outlined by Cachat et al. [40].

#### 4.6.2. Novel Approach Test (NAT)

To analyze the locomotor and anxiety-like behaviors of zebrafish in the face of novel stimuli, the NAT was used [28]. The test was performed in a 34 cm diameter and 15 cm high opaque white plastic cylinder, with water from the fish housing environment, changed after each series of tests. The arena was divided into two zones: the inner zone (a 10 cm diameter circle) and the outer zone (the thigmotaxis zone, located on the wall of the arena). The water during testing was maintained at 27 ± 1 °C and changed after each experimental group. The locomotion of the fish was recorded for 5 min, measuring the time spent in each zone, the distance traveled, the immobility time, and the latency. The object chosen for the test was a 5 cm high multi-colored Lego figurine, according to previous studies [28].

#### 4.6.3. Light–Dark Test (LDT)

The LDT is based on the attraction of adult zebrafish to a dark environment, highlighting the internal conflict between the desire to remain in “safe” areas (the dark area, where melanophores help reduce light reflection and the risk of being detected by predators) and the desire to explore new environments. Despite this conflict, zebrafish exhibit a preference for dark areas and show light avoidance behavior. The LDT was conducted in a tank measuring 55 cm in length, 9.5 cm in height, and 9.5 cm in width, divided into two equal sections—one white and one black—to create a contrast between the light and dark areas, as previously described by Facciol et al. [41]. The tank was placed in a white space to minimize external visual stimuli. The water temperature was maintained at 27 ± 1 °C and the water was changed between experimental groups. One fish from each group was placed in the center of the arena, and locomotor behavior was recorded for 5 min. After every 7th fish, the tank was rotated 180° to prevent potential distortions.

#### 4.6.4. Y-Maze

The Y-maze test was used to study the behavior of zebrafish toward an unfamiliar environment [42]. The fish’s position in the unfamiliar arm of a Y-shaped tank (dimensions: 25 × 8 × 15 cm for each arm, capacity 5 L) served as a memory cue. The tank included three arms: “start” (the starting arm), “other” (which was always accessible), and “novel” (which was closed during training and opened during the test session). During the training session (5 min), the fish was placed in the start arm, with the novel arm being closed. After one hour, the test session (5 min) began, and the fish was returned to the start arm, now with the novel arm open. The water temperature was maintained at 27 ± 1 °C and the water was changed between experimental groups. Locomotor activity was assessed by measuring the total distance traveled (m) and the turn angle (°), while the response to novelty was determined by the time spent in the novel arm, expressed as a percentage of the total time spent in the three arms.

#### 4.6.5. Novel Object Recognition (NOR)

NOR is widely used to assess memory performance in zebrafish [43]. In this test, zebrafish are subjected to an acclimation period of 5 min per day, for 3 consecutive days, in a new tank (30 × 30 × 30 cm), with 5 cm of water, without the presence of objects. On the fourth day, zebrafish are exposed to two identical objects (familiar objects, FO) for 10 min during the training phase. One hour after this phase, during the test phase, one of the FOs is replaced by a novel object (NO). The interaction between zebrafish and objects is observed for another 10 min. The water throughout the entire experimental period was maintained at 27 °C ± 1 °C and changed after each experimental group. The preference for the novel object is quantified by calculating the following ratio:

$$Preference\;(\%)=\frac{Time\;spent\;exploring\;the\;NO}{Time\;spent\;exploring\;the\;FO+Time\;spent\;exploring\;the\;NO}\times 100$$

### 4.7. Biochemical Parameter Testing

After behavioral testing, zebrafish were euthanized by rapid cooling (10 min in cold water at 2–4 °C). Brains were dissected and homogenized using a Mikro-Dismembrator U (Sartorius, New York, NY, USA) with 3 mm diameter magnetic beads (Sartorius Stedim Biotech GmbH, Goettingen, Germany) in 0.1 M potassium phosphate buffer (pH 7.4) containing 1.15% KCl. Samples were centrifuged at 960× *g* for 15 min, and the supernatant was used to assess antioxidant enzyme activities and oxidative metabolite levels.

#### 4.7.1. Determining Acetylcholinesterase (AChE) Activity

AChE activity in the whole zebrafish brain was measured using Ellman’s photometric method [44]. The substrate acetylthiocholine iodide (ATCh) and the reagent 5,5′-dithio-bis-2 nitrobenzoic acid (DTNB) were combined in a phosphate buffer (pH 7.4). Enzyme activity was assessed spectrophotometrically at 412 nm, following the formation of a yellow color generated by the reaction between thiocholine and DTNB. The results were expressed in nmol ATCh/min/mg protein, and the protein content was quantified by the Bradford method [45].

#### 4.7.2. Determining Superoxide Dismutase (SOD) Activity

SOD activity was measured as previously described by Artenie et al. [46]. The samples, containing a final mixture of 0.067 M potassium phosphate solution, enzyme extract, 0.1 M EDTA solution, 0.12 mM riboflavin, and 1.5 mM NBT, were read at 560 nm. The enzyme activity was correlated with the protein concentration in the extract, with specific SOD activity expressed in enzyme units per mg of protein, as determined by the Bradford method [45].

#### 4.7.3. Determining Catalase (CAT) Activity

CAT activity was evaluated according to Sinha’s protocol [47]. The reaction tubes contained 125 µL of enzyme homogenate and 125 µL of 0.16 M H_2_O_2_ substrate solution. After 180 s, the reaction was terminated by adding 500 µL of potassium dichromate–glacial acetic acid solution, and the tubes were incubated at 95 °C for 10 min. Following incubation, the tubes were centrifuged at 14,000 rpm for 5 min, and the absorbance of the supernatant was measured at 570 nm. Enzyme activity was expressed as µmoles of H_2_O_2_ consumed/min/mg protein.

#### 4.7.4. Determining Glutathione Peroxidase (GPX) Activity

GPX activity was assessed as previously described by Fukuzawa and Tokumura [48]. In 1.5 mL tubes, enzyme extract, 0.25 M phosphate buffer, 25 mM EDTA, and 0.4 M NaN_3_ were added. After 10 min of incubation at 37 °C, GSH and H_2_O_2_ were added, and the reaction continued for another 10 min. The reaction was stopped with metaphosphoric acid, and the samples were measured for absorbance at 412 nm, after centrifugation and addition of reagents. Enzyme activity was expressed in units per mg of protein.

#### 4.7.5. Determining Carbonylated Protein Contents

The level of carbonylated proteins was determined using the method of Luo and Wehr [49], which is based on the interaction of 2,4-dinitrophenylhydrazine with protein residues, yielding 2,4-dinitrophenylhydrazones, which can be measured at 370 nm, compared to a mixture of GuHCl and KH_2_PO_4_. The results obtained were expressed in nmol DNPH per mg protein.

#### 4.7.6. Malondialdehyde (MDA) Level

The MDA level was measured using the method of Ohkawa et al. [50]. The tubes contained 200 µL of supernatant, 1 mL of 50% trichloroacetic acid, 1 mL of 26 mM thio-barbituric acid, and 0.1 M HCl. After vortexing, the samples were heated at 95 °C for 20 min and then cooled on ice for 5 min. After centrifugation at 960× *g* for 10 min, the supernatant was measured at 532 nm. The results were expressed in nmol/mg protein.

### 4.8. Data Analysis

Results were expressed as mean ± standard error of the mean (SEM). All behavioral and biochemical data were first assessed for normality using the Shapiro–Wilk test. Group differences were assessed using two-way analysis of variance (ANOVA) followed by the Tukey HSD post hoc test, with treatment and experimental condition as a factors. Statistical significance was defined as *p* < 0.05. All statistical analyses were conducted using GraphPad Prism 9.4 software (GraphPad Software, Inc., San Diego, CA, USA). Correlations between behavioral scores, enzymatic activities, and lipid peroxidation were evaluated using the Pearson correlation coefficient (r).

## 5. Conclusions

The results of this study suggest that SMEE has significant neuroprotective and anxiolytic effects, potentially mediated through modulation of the cholinergic system, reduction in oxidative stress, and other neurotransmission mechanisms. The beneficial effects observed in zebrafish highlight its therapeutic potential in managing cognitive dysfunction and anxiety-related disorders. However, certain limitations must be considered. This study was conducted solely on zebrafish, posing challenges in extrapolating findings to mammals and humans. Additionally, the precise molecular mechanisms underlying the observed benefits require further elucidation. Variability in the phytochemical composition of SMEE may affect reproducibility, necessitating standardization efforts. Furthermore, potential adverse effects of chronic SMEE administration were not explored, requiring comprehensive toxicological evaluations.

Future studies should focus on validating these findings in higher animal models, such as rodents, to confirm the therapeutic efficacy of SMEE in conditions closer to human physiology. Detailed characterization of active compounds, along with molecular studies (proteomics, metabolomics), will be essential for understanding their mechanisms of action. Investigating the pharmacokinetic and pharmacodynamic interactions of SMEE, along with bioavailability studies, will provide crucial insights into its feasibility as a therapeutic agent. Finally, preliminary clinical trials could offer further evidence on the efficacy and safety of SMEE in treating cognitive and anxiety disorders, paving the way for potential medical applications.

## Figures and Tables

**Table 1:** Amount of phenolic compounds in the ethanolic extract of *Solanum macrocarpon* (SMEE), retention times, and linear relationships between peak areas and concentrations.

Sample	Compounds	Retention Time (min)	Maximum Absorbance (nm)	Amount (mg/g, Mean ± SD)	Standard Curve	R^2^
SMEE	Chlorogenic acid	15.595	320	25.46 ± 0.10	y = 28.919x + 54.92	0.9974
	Rutin	30.000	260	7.55 ± 0.10	y = 36.127x + 150.42	0.9993

**Table 2:** The pharmacokinetic and toxicological properties of the analyzed compounds (SCOP, GAL, chlorogenic acid, and rutin).

Property	Compound Model Name	SCO	GAL	Chlorogenic Acid	Rutin	Unit
Absorption	Intestinal absorption (human) (low < 30%, high > 30%)	72.626	94.994	36.377	23.446	Numeric (% Absorbed)
	Skin permeability (low logKp > −2.5, high logKp < −2.5)	−4.097	−3.75	−2.735	−2.735	Numeric (log Kp)
	P-glycoprotein substrate	Yes	No	Yes	Yes	Categorical (Yes/No)
	P-glycoprotein I inhibitor	No	No	No	No	Categorical (Yes/No)
	P-glycoprotein II inhibitor	No	No	No	No	Categorical (Yes/No)
Distribution	VDss (human) (low log VDss < −0.15, high VDss > 0.45)	0.583	0.89	0.581	1.633	Numeric (log L/kg)
	Unbound fraction (human)	0.414	0.36	0.658	0.187	Numeric (Fu)
	BBB permeability (log BB > 0.3 cross BB, log BB < 0.1 do not cross BB)	−0.043	0.081	−1.407	−1.899	Numeric (BB log)
	CNS permeability (log PS > −2 penetrates CNS, log PS < −3 does not penetrate)	−3.031	−2.511	−3.856	−5.178	Numeric (PS log)
Metabolism	CYP3A4 substrate	Yes	Yes	No	No	Categorical (Yes/No)
	CYP1A2 inhibitor	No	No	No	No	Categorical (Yes/No)
Excretion	Total clearance	1.096	0.991	0.307	−0.369	Numeric (log mL/min/kg)
	Renal OCT2 substrate	No	Yes	No	No	Categorical (Yes/No)
Toxicity	hERG blockers	0.19	0.458	0.025	0.008	Numeric
	hERG blockers (10 µm)	0.418	0.625	0.093	0.263	Numeric
	DILI	0.104	0.215	0.291	0.937	Numeric
	AMES toxicity	0.158	0.559	0.386	0.756	Numeric
	Carcinogenicity	0.015	0.726	0.225	0.047	Numeric
	Human hepatotoxicity	0.895	0.795	0.543	0.406	Numeric
	Drug-induced nephrotoxicity	0.403	0.802	0.441	0.148	Numeric
	Drug-induced neurotoxicity	0.753	0.744	0.009	0.0	Numeric
	Hematotoxicity	0.16	0.505	0.028	0.023	Numeric
	Genotoxicity	0.677	0.739	0.243	0.868	Numeric
	RPMI-8226 immunotoxicity	0.043	0.097	0.016	0.098	Numeric
	A549 cytotoxicity	0.058	0.069	0.203	0.86	Numeric

## Data Availability

The data are contained within this article.
